# Effects of increasing narasin doses on feedlot performance, feeding behavior, carcass traits, and rumen-cecum morphometrics in Nellore cattle

**DOI:** 10.1371/journal.pone.0344240

**Published:** 2026-03-18

**Authors:** Leandro Aparecido Ferreira da Silva, Daniel Moretto Casali, Ana Laura Januário Lélis, Tiago Leiva, Murilo Chuba Rodrigues, José Paulo Roman Barroso, Pedro Veloso Facury Lasmar, Giovanna Lombardi de Oliveira Araújo, Gercino Ferreira Virgínio Júnior, Johnny Maciel de Souza, Danilo Domingues Millen

**Affiliations:** 1 School of Veterinary Medicine and Animal Science, São Paulo State University (UNESP), Botucatu, São Paulo Brazil; 2 Elanco Animal Health, São Paulo, São Paulo, Brazil; 3 School of Agricultural and Veterinary Sciences, São Paulo State University (UNESP), Jaboticabal, São Paulo Brazil; University of Agriculture Faisalabad, PAKISTAN

## Abstract

Narasin is an ionophore commonly used to improve energy efficiency and feed utilization in beef cattle. This study evaluated the impact of increasing levels of narasin on performance, feeding behavior, carcass traits, and ruminal and cecum morphometrics of Nellore cattle in feedlot. One hundred twenty-eight Nellore yearling bulls (393 ± 24 kg) were blocked by initial body weight (BW), allocated in 32 pens (4 bulls/pen). Pens were randomly assigned to one of four treatments: 0 (control), 13, 20, or 27 ppm of narasin. After a 14-day adaptation, animals were fed a high-concentrate diet (87% concentrate, dry matter basis) for 98 days. Narasin was not included in the diet from day 109 to day 111 due to the withdrawal period required by the active compound. Animals were slaughtered on day 112. A significant linear effect of narasin was observed on BW (P = 0.02) and average daily gain (P = 0.01) during the first 28 days, with improved feed efficiency at 20-ppm (P = 0.03). These effects did not persist over the full 111 d period. Cattle receiving 20-ppm of narasin had longer meal durations on day 70 (P = 0.05). A quadratic effect was observed on the thickness of fat at the 12th rib (P = 0.05), with the highest mean value observed at 20 ppm. The 13-ppm level increased the rumen papillae size (P = 0.02), while higher levels reduced the number of goblet cells in the cecum (P = 0.01). Narasin supplementation enhanced early feedlot performance and carcass fat deposition without negatively affecting dry matter intake. Narasin can be used in feedlot diets from 13 to 27 ppm, with 20 ppm being the most effective level for improving performance and carcass quality.

## Introduction

Concentrate-rich diets often lead to high production of ruminal short-chain fatty acids (SCFA), which can improve feedlot performance but may also cause metabolic disorders such as ruminal acidosis if ruminal fermentation is not properly managed [1–3]. Feed additives, particularly ionophores, are widely used to modulate ruminal fermentation, improving energy utilization and reducing the risk of metabolic disturbances [4–7].

An ionophore that has received limited attention in beef cattle is narasin, commonly used in poultry for its coccidiostatic properties [8] and as a growth promoter in swine [9]. Narasin, produced by *Streptomyces aureofaciens*, selectively inhibits Gram-positive bacteria, shifting ruminal fermentation toward higher propionate and lower acetate concentrations [10,11]. Early in vitro studies demonstrated that narasin more effectively increases propionate and reduces lactic acid accumulation than other ionophores, including monensin and lasalocid [12,13] Recent evidence indicates that narasin enhances feedlot performance without affecting dry matter intake (DMI) [10,14]. Most studies have examined narasin in combination with monensin in high-concentrate diets [15,16]; however, no studies have evaluated the effects of different narasin doses on feedlot diets for Nellore cattle in Brazil, which typically have lower energy content than U.S. finishing diets (1.27 vs. 1.50 Mcal of net energy for gain/kg of DM in Brazil vs. the USA, respectively) [1,5].

Furthermore, changes in ruminal fermentation can alter epithelial exposure to SCFA, and ionophores may indirectly affect structural adaptations in the gastrointestinal tract. This connection is important under high-concentrate feeding conditions because the shift in ruminal fermentation or excessive starch entering the hindgut may affect both ruminal absorptive capacity and cecal integrity. A recent study on Nellore cattle showed that feed additives capable of modulating fermentation also increased ruminal absorptive surface area and reduced cecal lesion scores [17], highlighting the importance of evaluating rumen and cecum morphometrics when assessing physiological responses to narasin doses.

A dose of 13 ppm of narasin is recommended for cattle grazing on or fed high-forage diets [14]. However, the optimal dose of narasin for feedlot cattle has not yet been determined. Therefore, this study aimed to evaluate the effects of increasing inclusion levels of narasin in feedlot diets on performance, feeding behavior, carcass characteristics, and ruminal and cecal morphometrics of Nellore yearling bulls.

## Materials and methods

Animal care and handling used in this experiment adhered to the guidelines of the Animal Use Ethics Committee (CEUA) and were approved by the Ethics Committee on Animal Use of São Paulo State University (UNESP), Dracena campus Brazil (Protocol CEUA 002/2022).

### Animals and experimental facility

The study was conducted at the UNESP feedlot, Dracena campus, Brazil. A total of 128, 18-month-old Nellore bulls (393 ± 24 kg), with an average frame score of 5 based on NASEM growth standards [17], were randomly allocated into 32 pens (n = 4 animals per pen; providing 1.5m of linear bunk space and 18 m^2^ of pen space per animal), which were considered the experimental units for this study.

### Experimental design

The experimental design was a completely randomized block, using the initial body weight (BW) as the block criteria. The experimental treatments were based on narasin doses and were applied randomly per pen as follows: treatment 1: 0 ppm of narasin (control); treatment 2: 13 ppm of narasin; treatment 3: 20 ppm of narasin; and treatment 4: 27 ppm of narasin. Each treatment had eight replicates, and all animals began and concluded the study on the same days.

The 13 ppm treatment is commonly used in supplements for grazing systems [11,18,19]. Since feedlot diets have a higher energy content than forage diets, the tested doses were equal to or greater than the usual dose for grazing animals.

### Management, feeding, and animal handling

Before starting the study, all yearling bulls were identified, weighed, dewormed, and vaccinated for viral and bacterial diseases (tetanus, bovine viral diarrhea virus, 7-way Clostridium sp.; Cattlemaster, Zoetis, New York, NY). The study lasted for 112 days, which included a 14-day adaptation period. The adaptation protocol utilized a step-up method and involved three adaptation diets. The initial diet consisted of 75% concentrate, with 4% concentration increases between each adaptation phase. Tifton hay served as the sole source of forage and was gradually removed from the diets throughout the adaptation period. The transition from adaptation diets to the finishing diet was executed as follows: 5 days on adaptation 1, 4 days on adaptation 2 (79% concentrate), and 5 days on adaptation 3 (83% concentrate). The finishing diet contained 87% concentrate ingredients (Table 1).

**Table 1:** Feed ingredients and chemical composition of the experimental diets fed to the yearling bulls during the adaptation and finishing phases.

Diets, % DM	Adaptation 1	Adaptation 2	Adaptation 3	Finishing
**Concentrate**	75.0	79.0	83.0	87.0
**Ingredients**				
Tifton hay	15.0	11.0	7.0	3.0
Peanut hulls	10.0	10.0	10.0	10.0
Finely-ground corn	51.0	56.0	61.0	66.0
Soybean meal	10.0	8.0	6.0	4.0
Cottonseed cake	11.0	12.0	13.0	14.0
Urea (conventional)	0.5	0.5	0.5	0.5
Mineral supplement^3^	2.5	2.5	2.5	2.5
**Nutritional Content**				
TDN^1^	78.0	80.0	81.0	82.0
Crude protein	15.8	15.1	14.4	13.8
Ether extract	4.0	4.1	4.2	4.3
Neutral detergent fiber	33.9	31.7	29.6	27.5
peNDF^2^	23.0	21.0	18.0	16.0
NE_g_, Mcal/kg	1.12	1.15	1.19	1.23
Ca	0.52	0.50	0.48	0.47
P	0.41	0.41	0.41	0.42

^1^ Total digestible nutrients. ^2^Physically effective neutral detergent fiber. ^3^Composition per kg of dry matter: Calcium 160 g, Phosphorus 22 g, Sodium 70 g, Potassium 40 g, Magnesium 35 g, Sulfur 25 g, Cobalt 30 mg, Copper 450 mg, Iodine 25 mg, Manganese 850 mg, Selenium 5 mg, Zinc 1350 mg, Chromium 15 mg, Vitamin A 60,000 IU, Vitamin D 8,000 IU, Vitamin E 480 IU The additive were added to the premix to make a total of 13, 20 or 27 ppm of Narasin (Zimprova, Elanco, USA).

The experimental diets were formulated according to the LRNS (Large Ruminant Nutrition System; [20], level 2. Feed ingredients were mixed in a truck-mounted mixer. The cattle were fed ad libitum twice daily throughout the study, and the feed was provided in a bunk feeder (6m; 1.2m per animal per pen) at 08:00 (45% of the total ration) and 16:00 (55% of the total ration). The inclusion levels of narasin in the finishing diets were established by batch calculation based on diet dry matter intake and target ppm concentrations. Narasin was premixed with soybean meal as a carrier and top-dressed onto the diets, followed by immediate and thorough mixing in the bunk feeder to ensure uniform distribution.

Experimental diets and ingredients were sampled weekly [21]. The DM (method 930.15), ether extract (method 920.39), crude protein (method 990.02), and ash (method 942.05) were determined according to the Association of Official Analytical Chemists [22]. Neutral detergent fiber (NDF) was determined according to the procedures of Van Soest et al. [23]. The amount of feed provided per pen was adjusted daily based on the orts from the previous day before the morning feed delivery at 08:00. Refused feed was discarded daily. The yearling bulls had free access to the water trough, which measured 0.89 × 1.00 × 2.00 m. The DMI was measured for each pen by weighing the feed offered daily and the orts before the next morning’s feeding, approximately 16 hours after the previous feed delivery. The DM content of the total mixed diet was determined daily, and a composite sample of the orts for each pen was collected weekly.

### Performance

The animals were weighed on days 0, 16, 28, 56, 84, and 111 of the experimental period. The initial and final BW were measured after 16 hours of fasting from solid feeds. In the other BW assessments, the shrunk BW was determined following a 4% BW deduction. The DMI for each pen was measured daily and expressed in both kilograms and as a percentage of BW. Average daily gain (ADG) was calculated cumulatively throughout the study. Using ADG and DMI data, the gain-to-feed ratio (G:F) for each pen was calculated [24]. The DMI variation was calculated as the difference in intake between two consecutive days and expressed in both kilograms and as a percentage of variation [24]. To estimate the net energy for maintenance (NEm) and the net energy for gain (NEg) of the experimental diets, the methods described by Zinn and Shen [25] were used.

### Feeding behavior

The feeding behavior data were collected on day 70 of the study, following the methodology adapted from Robles et al. [26]. Day 70 was selected to ensure the animals had adapted to the finishing diet and because the DMI should be stable by this point, making this measurement representative of the finishing period. Feeding behavior data were recorded every 5 minutes over 24 hours for each animal, including the following parameters: time spent eating, time spent ruminating, time spent resting, all expressed in minutes, and the number of meals per day. A meal was defined as the uninterrupted time that the bull remained at the feed bunk consuming feed, using a presence-based criterion [27]. Additionally, DM and NDF intake for each pen were measured on the day the data were collected. Meal duration (in minutes) was calculated by dividing the time spent eating by the number of meals per day. The DMI per meal (in kilograms) was determined by dividing the total DMI by the daily number of meals per pen. Furthermore, data on time spent eating and ruminating were used to calculate the DM and NDF intake rates and the ruminating rates, all expressed in minutes per kilogram of DM or NDF.

### Particle sorting

Samples of the diets and orts were collected on day 70 to determine the particle size distribution using the Penn State Particle Size Separator, as described by Heinrichs and Kononoff [28]. Particle sorting was calculated as follows: n intake/ n predicted intake, where n represents the fraction of particles larger than 19 mm (long), 8 mm (medium), 1.18 mm (short), and smaller than 1.18 mm (fine). A particle sorting value equal to 1 indicates no sorting, values less than 1 indicate refusals (sorting against), and values greater than 1 indicate preferential consumption (sorting for), as described by Leonardi and Armentano [29].

### Carcass characteristics

Due to the withdrawal period, narasin was excluded from the finishing diet on days 109, 110, and 111. After the final weighing on day 111, the yearling bulls were transported approximately 50 km to a commercial slaughter facility and slaughtered the following day in accordance with standard industry practices and Brazilian animal welfare regulations [25]. After slaughter, the hot carcass weight (HCW) was recorded following the removal of kidney, pelvic, and heart fat, and the dressing percentage was calculated by dividing the HCW by the final BW.

The 12th rib fat thickness (RFT), fat thickness of the biceps femoris (P8), rib-eye area (REA), and marbling were measured using ultrasound at the beginning (day 0) and end of the experimental period, following the method described by Perkins et al. [30]. The daily gains of RFT, P8, and REA were calculated as the difference between the two measurements divided by the number of days on feed (111 days). Images were collected with an Aloka SSD-1100 Flexus RTU (Aloka Co. Ltd., Tokyo, Japan), using a 17.2 cm, 3.5 MHz transducer probe.

### Rumen and cecum morphometrics

#### Rumen morphometrics.

Lesions in the ruminal compartment (rumenitis) were examined after eviscerating the yearling bulls and thoroughly washing out all the contents. The ruminal epithelium was classified based on the incidence of lesions (rumenitis and parakeratosis) and other abnormalities (e.g., papillae clumped) following the methodology described by Bigham and McManus [31], and using a scale from 0 (no lesions and abnormalities noted) to 10 (ulcerative lesions throughout the rumen). Scores for ruminal lesions were independently assigned by two trained observers and subsequently averaged; however, inter-observer reliability was not statistically assessed. The lesions observed were not experimentally induced but represented naturally occurring responses to the high-concentrate diet, which commonly predisposes feedlot cattle to subacute acidosis and epithelial damage.

For macroscopic ruminal morphometrics, a 1 cm^2^ fragment was collected from the dorsal cranial sac of each rumen and preserved in a 70% alcohol solution for future morphometric measurements, following the method described by de Resende-Junior [32]. The number of papillae per square centimeter of the ruminal wall (NOP) was determined manually. Twelve papillae were randomly collected from each fragment and digitized, and the mean papillae area (MPA) was calculated using an image analysis system (Image Tool, version 2.01 alpha 4, UTHSCSA Dental Diagnostic Science, San Antonio, TX). The absorptive surface area (ASA) of the rumen wall in square centimeters was calculated as follows: 1 + (NOP × MPA) – (NOP × 0.002), where 1 represents the 1-cm^2^ fragment collected, and 0.002 is the estimated basal area of the papillae in square centimeters. The papillae area, expressed as a percentage of ASA, was calculated as follows: (NOP × MPA)/ASA × 100.

**Fig 1:**
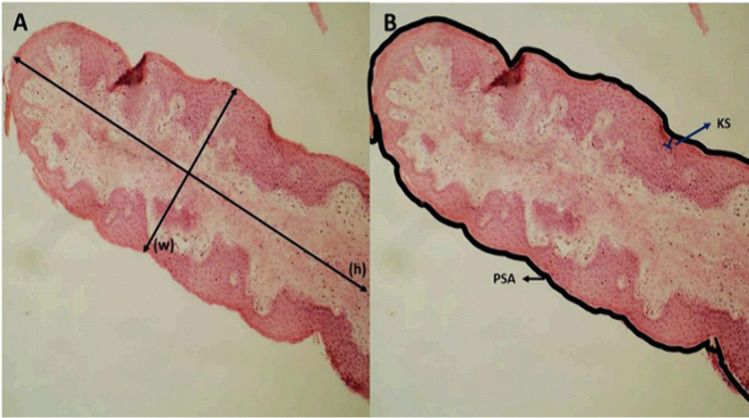
Representative histological section of ruminal papillae illustrating the measurements performed for height (h), width (w), papillae surface area (PSA), and keratinized layer thickness (KS).

Similarly, a 1 cm^2^ fragment from the ventral cranial sac of each rumen was collected for microscopic ruminal morphometric evaluation using the histological technique. The histological sections were stained with hematoxylin and eosin, embedded in paraffin, and sectioned [33]. Histological measurements, including papillae height, width, surface area, and keratinized layer thickness, were performed on four papillae per animal using computer-aided light microscopy analysis. Representative histological sections illustrating the measurement procedures for papillae height (h), width (w), papillae surface area (PSA), and keratinized layer thickness (KS) are shown in Fig 1.

#### Cecum morphometrics.

The evaluation of lesions in the cecum was conducted after the cattle were eviscerated, and the cecal compartment was washed. The epithelium of the cecum was classified based on the presence of inflammation, lesions, and petechiae on the cecal wall, using a scale from 0 (no lesions) to 10 (severe lesions), adapted from Bigham and McManus [31]. Similar to rumen lesion evaluations, scores for cecal lesions were independently assigned by two trained observers and then averaged. No inter-observer reliability was statistically assessed.

For cecum morphometrics, a 1-cm^2^ fragment was collected from the center of the cecum compartment for histological evaluation and preserved in a 4% buffered paraformaldehyde solution for future analysis [34]. For histological analysis of the cecal epithelium, tissue samples were dehydrated, embedded in paraffin wax, sectioned at eight μm, and stained with hematoxylin and eosin [35]. Histological measurements, including crypt depth and the number of enterocytes and goblet cells, were determined in 10% of the total crypts per sample per animal using a Leica Qwin Image Analyzer within a Leica electron light microscope [35].

### Statistical analysis

Data were analyzed using a randomized complete block design with the MIXED procedure of SAS (v. 9.2; SAS Institute Inc., Cary, NC, USA). Blocks were included as random effects in the model, while treatments were considered fixed effects. Where appropriate, initial BW and ultrasound measurements served as covariates in the statistical model. The CONTRAST option in SAS was used to examine the following effects: 1) the linear effect of narasin doses, 2) the quadratic effect of narasin doses, and 3) the cubic effect of narasin doses. The ORPOL function in SAS generated the orthogonal contrasts for the unevenly spaced doses of narasin. Statistical significance was set at P ≤ 0.05.

## Results

### Performance

The results of feedlot performance are presented in Table 2. During the first 28 days on feed, a significant linear increase was observed in BW (P = 0.02) and ADG (P = 0.01), along with improved G:F (P = 0.03) as narasin dose increased. In contrast, no significant effects of narasin were detected for these performance variables during later periods or over the entire feeding period (P > 0.05). Quadratic effects (P ≤ 0.05; Table 2) were observed for DMI from 0 to 84 days and from 0 to 111 days on feed, where cattle receiving 13 and 20 ppm of narasin showed greater feed intake, expressed either in kg (P < 0.05) or as a percentage of BW (P < 0.02). No significant effects of narasin dose were found for DMI variation or net energy variables (P > 0.05).

**Table 2:** Feedlot performance of Nellore yearling bulls fed diets containing increasing doses of narasin.

Item	Narasin (ppm)				SEM^1^	P value		
	0	13	20	27		L^2^	Q^3^	C^4^
**Body weight, kg**								
Day 0	393.20	392.31	392.82	393.32	8.33	0.96	0.52	0.87
Day 16	410.82	408.94	412.11	411.63	8.45	0.91	0.89	0.83
Day 28	423.95	424.07	426.75	430.65	1.92	**0.02**	0.17	0.93
Day 56	470.31	468.67	471.79	474.71	2.50	0.21	0.24	0.74
Day 84	506.19	508.17	511.97	514.49	3.79	0.11	0.71	0.81
Day 111	568.62	574.15	572.43	571.01	3.92	0.63	0.35	0.84
**DMI** ^**5**^ **, kg**								
0 to 16 days	9.00	9.18	9.17	9.07	0.12	0.57	0.37	0.96
0 to 28 days	9.30	9.48	9.74	9.52	0.18	0.14	0.55	0.46
0 to 56 days	9.99	10.20	10.33	10.14	0.14	0.21	0.23	0.42
0 to 84 days	10.33	10.69	10.57	10.38	0.15	0.70	**0.05**	0.79
0 to111 days	10.38	10.80	10.63	10.37	0.15	0.92	**0.02**	0.77
**DMI, % of BW**								
0 to 16 days	2.24	2.29	2.28	2.26	0.03	0.57	0.29	0.86
0 to 28 days	2.26	2.33	2.37	2.31	0.04	0.23	0.42	0.49
0 to 56 days	2.31	2.37	2.39	2.34	0.03	0.29	0.10	0.45
0 to 84 days	2.30	2.37	2.34	2.29	0.03	0.87	**0.02**	0.61
0 to 111 days	2.16	2.23	2.20	2.15	0.02	0.92	**0.01**	0.71
**ADG** ^**6**^ **, kg/d**								
0 to 16 days	1.10	1.04	1.21	1.15	0.13	0.66	0.83	0.45
0 to 28 days	1.11	1.11	1.21	1.35	0.07	**0.01**	0.15	0.94
0 to 56 days	1.38	1.35	1.41	1.46	0.04	0.18	0.21	0.77
0 to 84 days	1.35	1.37	1.42	1.45	0.04	0.07	0.73	0.83
0 to 111 days	1.58	1.63	1.62	1.60	0.04	0.66	0.40	0.81
**Gain-to-feed ratio**								
0 to 16 days	0.121	0.115	0.130	0.126	0.010	0.66	0.77	0.51
0 to 28 days	0.119	0.119	0.125	0.142	0.010	**0.03**	0.14	0.85
0 to 56 days	0.139	0.134	0.137	0.144	0.009	0.30	0.12	0.98
0 to 84 days	0.131	0.129	0.134	0.140	0.009	0.13	0.24	0.75
0 to 111 days	0.153	0.152	0.152	0.155	0.008	0.63	0.45	0.91
**DMI variation, %**	0.57	0.55	0.55	0.59	0.04	0.90	0.52	0.83
**DMI variation, kg**	5.69	5.28	5.43	6.00	0.40	0.69	0.20	0.92
**NEm obs** ^**7**^ **/NEm** ^**8**^	1.13	1.12	1.13	1.15	0.01	0.54	0.15	1.00
**NEg obs** ^**9**^ **/NEg** ^**10**^	1.18	1.16	1.17	1.19	0.02	0.54	0.15	1.00

^1^ Standard error of the mean; ^2^ Linear effect of the inclusion of narasin; ^3^ Quadratic effect of the inclusion of narasin; ^4^ Cubic effect of the inclusion of narasin; ^5^Dry matter intake; ^6^ Average daily gain; ^7^ Net energy for maintenance observed; ^8^Net energy for maintenance expected; ^9^Net energy for gain observed; ^10^Net energy for gain expected.

### Feeding behavior and particle sorting

The results of feeding behavior and particle sorting are shown in Table 3. A cubic effect (P = 0.05) was noted for meal length, indicating that Nellore cattle receiving diets with 20 ppm of narasin spent more time to finish a meal. As narasin doses increased, a quadratic effect (P = 0.03) was observed for DMI, peaking for cattle fed 13 ppm of narasin on the day of the feeding behavior evaluation. Different doses of narasin had no influence on other variables related to feeding behavior or particle sorting (P > 0.05).

**Table 3:** Feeding behavior and particle sorting of Nellore yearling bulls fed feedlot diets containing increasing doses of narasin.

Item	Narasin (ppm)				SEM^1^	P value		
	0	13	20	27		L^2^	Q^3^	C^4^
*Feeding behavior*								
Time spent resting, min	1161.49	1135.70	1169.50	1181.13	17.94	0.37	0.18	0.39
Time spent ruminating, min	135.14	161.18	116.66	123.69	14.71	0.34	0.27	0.09
Time spent eating, min	143.36	143.12	153.84	135.19	9.34	0.79	0.38	0.19
Meal length, min	10.30	10.08	11.10	9.97	0.41	0.95	0.45	**0.05**
Meals per day, n	13.97	14.20	14.03	13.59	0.77	0.75	0.61	0.98
DMI^5^, Kg	10.96	11.90	11.00	10.61	0.40	0.40	**0.03**	0.24
DMI per meal	0.80	0.86	0.81	0.79	0.05	0.82	0.34	0.55
ERDM^6^, min/kg of DM	13.14	12.07	14.14	12.94	0.84	0.78	0.79	0.10
RRDM^7^, min/kg of DM	12.41	13.65	10.81	11.76	1.36	0.49	0.65	0.19
NDF^8^ intake, Kg	3.619	4.130	3.730	3.950	0.34	0.59	0.58	0.35
ERNDF^9^, min/kg of NDF	43.18	37.55	42.44	34.88	3.55	0.29	0.82	0.13
RRNDF^10^, min/kg of NDF	39.42	43.15	33.57	32.39	5.17	0.25	0.41	0.38
*Particle sorting*								
Long	1.03	1.02	1.02	0.96	0.02	0.08	0.29	0.18
Medium	1.02	1.02	1.01	1.01	0.01	0.44	0.98	0.97
Short	1.00	1.00	0.99	1.00	0.00	0.88	0.56	0.09
Fine	0.99	0.99	1.00	0.99	0.01	0.45	0.67	0.50

^1^ Standard error of the mean; ^2^ Linear effect of the inclusion of narasin; ^3^ Quadratic effect of the inclusion of narasin; ^4^ Cubic effect of the inclusion of narasin; ^5^ Dry matter intake; ^6^ Eating rate of dry matter; ^7^ Rumination rate of dry matter; ^8^ Neutral detergent fiber; ^9^ Eating rate of NDF; ^10^ Rumination rate of NDF.

### Carcass characteristics

Results related to carcass characteristics are presented in Table 4. No significant effects of narasin doses were observed for most evaluated carcass variables (P > 0.05). A quadratic effect was noted for final 12th rib fat and 12th rib fat gain (P = 0.05), with cattle receiving 20 ppm of narasin showing greater fat deposition. It is important to note that this effect, similar to the early BW and ADG improvements observed during the first 28 days on feed, did not translate into overall differences in final body weight or cumulative performance at day 111, as indicated by the lack of significant differences in final BW (P = 0.63; Table 2).

**Table 4:** Carcass characteristics of Nellore yearling bulls fed feedlot diets containing increasing doses of narasin.

Item	narasin (ppm)				SEM^1^	P value		
	0	13	20	27		L^2^	Q^3^	C^4^
HCW^5^, kg	315.77	316.25	316.76	317.83	3.42	0.67	0.89	0.98
Dressing, %	55.53	55.06	55.28	55.64	0.36	0.90	0.27	0.88
Initial REA^6^, cm^2^	64.45	64.73	64.73	65.43	1.00	0.55	0.80	0.81
Final REA, cm^2^	93.71	95.57	93.90	92.99	1.17	0.48	0.26	0.64
REA daily gain, cm^2^	0.284	0.298	0.267	0.312	0.020	0.67	0.62	0.12
Initial 12th rib fat, mm	2.89	2.80	2.77	3.07	0.10	0.49	0.09	0.25
Final 12th rib fat, mm	5.94	6.24	6.77	5.86	0.25	0.60	**0.05**	0.07
12th rib fat daily gain, mm	0.029	0.032	0.036	0.030	0.002	0.39	**0.05**	0.16
Initial P8^7^, mm	4.69	4.58	4.57	4.82	0.14	0.68	0.21	0.68
Final P8, mm	8.86	9.35	9.58	9.07	0.37	0.50	0.26	0.60
P8 daily gain, mm	0.040	0.045	0.045	0.044	0.004	0.32	0.51	0.99
Initial marbling, %	1.82	1.75	1.88	1.97	0.15	0.44	0.46	0.75
Final marbling, %	2.82	2.76	2.84	2.74	0.06	0.50	0.69	0.20

^1^ Standard error of the mean; ^2^ Linear effect of the inclusion of narasin; ^3^ Quadratic effect of the inclusion of narasin; ^4^ Cubic effect of the inclusion of narasin; ^5^ Hot carcass weight; ^6^ rib-eye area; ^7^ fat thickness of the *Biceps femoris* (P8).

### Rumen and cecum morphometrics

The results of rumen and cecum morphometrics are presented in Table 5. The increasing doses of narasin did not affect the rumenitis scores and rumen macroscopic variables (P > 0.05) of Nellore cattle fed high-concentrate diets. However, quadratic (P = 0.02) and cubic (P = 0.03) responses were observed for papillae height and width, respectively, with cattle receiving 13 ppm of narasin exhibiting the largest and thickest rumen papillae. For cecum morphometrics, as the narasin dose increased, there was a linear reduction (P = 0.01) in the total number of Goblet cells and ruptured Goblet cells, along with a linear increase (P = 0.03) in the crypt depth/Goblet cell ratio. No further effects were observed (P > 0.05) in the cecum in response to the increasing doses of narasin.

**Table 5:** Rumen and cecum morphometrics of Nellore yearling bulls fed feedlot diets containing increasing doses of narasin.

Item	Narasin (ppm)				SEM^1^	P value		
	0	13	20	27		L^2^	Q^3^	C^4^
*Lesions*								
Rumenitis score	1.14	0.82	0.94	1.19	0.19	0.95	0.15	0.90
Cecum lesions score	1.56	1.55	1.42	1.14	0.33	0.36	0.57	0.96
*Rumen macroscopic variables*								
Mean papillae area, cm^2^	0.50	0.49	0.46	0.43	0.04	0.34	0.56	0.88
Number of papillae, n	57.27	58.18	63.13	56.71	2.62	0.63	0.27	0.26
ASA^5^, cm^2^	29.45	29.61	30.60	25.22	2.61	0.46	0.26	0.51
Papillae area, % of ASA	96.66	96.88	96.77	96.41	0.30	0.64	0.26	0.96
*Rumen microscopic variables*								
Papillae height, cm	2.17	2.93	2.34	2.25	0.20	0.95	**0.02**	0.10
Papillae width, mm	0.32	0.39	0.30	0.31	0.02	0.48	0.08	**0.03**
Papillae surface area, cm^2^	1.04	1.14	1.11	1.04	0.09	0.97	0.30	0.95
Keratinized layer thickness, μm	5.76	6.92	5.98	6.27	0.46	0.60	0.26	0.17
*Cecum microscopic variables*								
Crypt depth, μm	51.69	49.57	50.48	45.13	2.75	0.15	0.49	0.43
Enterocytes, n	20.15	19.38	18.68	16.78	1.44	0.12	0.54	0.85
Goblet cells, n	2.14	2.34	1.79	1.45	0.23	**0.01**	0.17	0.50
Ruptured Goblet cells, n	1.01	0.71	0.69	0.64	0.08	**0.01**	0.27	0.65
Crypt depth/Enterocytes	2.69	2.58	2.75	2.73	0.16	0.74	0.67	0.53
Crypt depth/Goblet cells	25.28	25.12	32.10	33.89	3.45	**0.03**	0.47	0.50

^1^ Standard error of the mean; ^2^ Linear effect of the inclusion of narasin; ^3^ Quadratic effect of the inclusion of narasin; ^4^ Cubic effect of the inclusion of narasin; ^5^ Absorptive surface area.

## Discussion

Feeding behavior and particle sorting were evaluated to explore potential mechanisms underlying narasin’s effects on nutrient utilization and ruminal fermentation. Ionophores like narasin can influence feed intake patterns and passage rates, which in turn affect ruminal stability, SCFA profiles, and nutrient absorption [14,19]. Measuring meal duration, eating rate, and sorting behavior provides insight into how narasin modulates feed utilization and contributes to observed differences in performance and carcass traits.

There are few studies on the use of narasin in concentrate-based diets [16] and its effects during specific periods of the cattle finishing process and in lambs [36]. Consequently, the impact of narasin during nutrient-deficit periods and the finishing phase in feedlots remains poorly understood. Given the limited research on these subjects, this study was conducted to determine the optimal dose of narasin for Nellore cattle consuming a feedlot diet.

In the present study, a significant effect of narasin on cattle BW and ADG was observed during the first 28 days, supporting the findings of Gobato et al. [19], who evaluated the effect of narasin supplementation in Nellore steers on pasture. Gobato et al. reported that supplementation with 13 ppm of narasin improved animal performance, consistent with results from other studies [11,14]. However, no significant effects of narasin were detected on BW, ADG, or G:F over the entire feeding period. This outcome does not imply that narasin was ineffective, as early improvements in performance may reflect transient alterations in ruminal fermentation and nutrient utilization that did not persist over time due to adaptation of the rumen microbiota to the high-concentrate diet. The yearling bulls receiving 20 ppm of narasin spent more time eating a meal; however, this did not influence the total DMI. Although the measurement of feeding behavior was only performed on day 70, this seems to be a limitation; however, that day was carefully chosen to ensure a proper representation of the finishing period. Additionally, cattle consuming 20 ppm of narasin showed greater fat deposition in the 12th rib, which may result from enhanced ruminal stability due to a slower DMI. This greater stability was also observed in the companion study involving cannulated animals conducted by Lelis et al. [37], in which bulls fed 20 ppm of narasin showed an increase in acetate, butyrate, and NH_3_ concentrations, along with a reduction in propionate concentration, as well as a smaller ruminal pH area < 5.6. Increased fat deposition contributes to meat quality and carcass weight, making fat accumulation an essential factor in improving growth performance [38]. However, no significant effect on HCW was observed in this study. This suggests that the improvement in fermentation patterns observed with narasin supplementation in ruminants [11] may be associated with more efficient dietary energy utilization by reducing energy losses from inefficient processes such as methane production [39], thereby enhancing feed efficiency. Studies investigating the effects of ionophores on carcass traits are limited and often yield conflicting results. Sardinha et al. [40] evaluated the inclusion of narasin in lamb diets and found that supplementing 13 ppm of narasin effectively increased subcutaneous fat thickness. For diets fed to feedlot cattle, however, the dose of narasin should be increased to 20 ppm to stimulate greater fat deposition. Moreover, the increased fat deposition observed in this study for animals fed 13 and 20 ppm of narasin may explain the lack of a positive effect on feedlot performance overall, as higher fat content in the carcass leads to greater maintenance needs for the animals. Additionally, subcutaneous fat is a thermal insulator, preventing rapid carcass cooling and consequently preventing sarcomere shortening, leading to tougher meat [41].

Another characteristic of narasin observed in this study was its lack of negative effect on DMI compared to the control treatment. Furthermore, recent studies in the literature have shown that narasin helps maintain DMI in diets with high NDF content [14] and high energy content [19]. Unlike other ionophores, narasin does not seem to decrease the DMI of beef cattle, regardless of diet type. Limede et al. [14] found that high-roughage diets with 13 ppm of narasin inclusion resulted in higher ADG, DMI, and BW over 140 days. The authors demonstrated that narasin had a significant effect on both DMI and ADG over a longer duration. However, in the study by [16], the various combinations of narasin and monensin in adaptation and finishing diets did not improve ADG or G:F compared to using a single ionophore throughout the period. Although narasin at 13 mg/kg of DM led to higher DMI during adaptation than monensin at 25 mg/kg DM, this had no effect on performance, digestibility, growth, or carcass characteristics in finishing cattle. Notably, the higher the energy content of a cattle diet, the lesser the impact of narasin on performance variables. Based on this fact, supplementing narasin in high-energy diets for feedlot cattle warrants further investigation.

Concerning the effects of narasin on the rumen and cecum epithelium, the increase in DMI for cattle given 13 ppm of narasin resulted in larger and thicker ruminal papillae, which may help reduce ruminal acidification despite a higher nutrient availability (on DM basis) for fermentation. Nagaraja et al. [12] noted in an in vitro study that narasin is more effective than other additives at inhibiting lactic acid production, which is closely linked to the decrease in ruminal pH [3]. This supports the appropriate size and width of the papillae, allowing for effective acid removal and stability of ruminal pH with narasin supplementation.

Ionophores such as narasin generally enhance the proportion of SCFA by increasing propionate production and reducing the C2:C3 ratio [10,14]. In the present study, ruminal stability was observed in association with slower DMI, and histological observations suggest that 20 and 27 ppm doses of narasin may have helped maintain cecal epithelial integrity, although direct measurements of cecal pH were not performed. This could indicate improved nutrient digestion in the upper gastrointestinal compartments, with nutrients not fermented in the rumen potentially undergoing partial fermentation in the cecum. Animals receiving either 13 ppm or no narasin exhibited a higher number of goblet cells, which protect colonocytes and support the mucus layer. Although direct mechanistic evidence in cattle is still scarce, data from mammalian studies indicate that SCFA derived from microbial fermentation stimulate mucin production and goblet-cell activity, enhancing mucosal barrier function and contributing to epithelial protection [42–44]. Similarly, the increased DMI in cattle fed 13 ppm of narasin may have accelerated liquid passage through the omasal–reticulum orifice, potentially enhancing large intestine fermentation. These hypotheses are supported by the histological observation of more goblet cells in the cecal epithelium of cattle receiving 13 ppm narasin; however, this did not correspond to a higher incidence of cecal lesions at the end of the finishing period.

Although the 27-ppm dose showed no significant difference in the data from the present study, in the companion study by Lelis et al. [37], the 27-ppm dose of narasin appears too high for feedlot cattle due to its negative effects on the ruminal environment (lower concentration of SCFAs and microbial diversity). Additionally, yearling bulls fed 27 ppm of narasin exhibited similar DMI to control bulls, indicating that the lower substrate amounts in both the rumen and the cecum may not have positively impacted these compartments compared to cattle consuming either 13 or 20 ppm of narasin.

## Conclusion

Narasin supplementation positively affected early feedlot performance and increased fat deposition on the 12th rib in Nellore bulls consuming 13 and 20 ppm. The results support the inclusion of narasin in feedlot diets at levels ranging from 13 to 27 ppm. Histological observations suggest that 20 ppm narasin may help maintain cecal epithelial integrity.
